# Postoperative complications and impaired long‐term survival—Is this causation or association?

**DOI:** 10.1002/ags3.12647

**Published:** 2022-12-14

**Authors:** Hideki Ueno, Hironori Tsujimoto

**Affiliations:** ^1^ Department of Surgery National Defense Medical College Tokorozawa Saitama Japan

**Keywords:** colorectal cancer, long‐term survival, postoperative complications, postoperative infections

Despite the recent developments in surgical technology and perioperative care, postoperative complications remain a major issue for surgeons because they cause adverse short‐term outcomes, including prolonged hospital stay, impaired quality of life, and even premature mortality. In addition to these adverse outcomes, infectious complications may increase the chance of recurrence of malignant disease, resulting in impaired long‐term survival. Conflicting results have been reported regarding this issue since the 1980s.^1^, ^2^

In the article accompanying this editorial, Matsuda et al.^3^ reported the results of a multicenter study conducted by the Japan Society for Surgical Infection that aimed to answer the thorny question, “Do postoperative infections complications really affect long‐term survival in colorectal cancer surgery?” The authors are commendable for undertaking such an important and challenging subject, even though their conclusions may be uncomfortable for surgeons. Tumor recurrence may result from postoperative infectious complications. Based on the retrospective analyses of 1817 patients with stage I–III colorectal cancer treated at 16 centers in Japan, the occurrence of postoperative infection after curative surgery was associated with impaired oncological outcomes, even after adjusting for differences in the patients' backgrounds. Specifically, patients who developed a postoperative infection had significantly worse outcomes in terms of cancer‐specific survival (CSS) and overall survival than those who did not develop postoperative infections (*p* < 0.001 for both). After conducting propensity matching for some clinical and oncological factors, 292 balanced pairs with significant CSS differences were identified. Patients, who did develop postoperative infections, were unfavored (*p* = 0.031). Based on the Kaplan–Meier curves, the severe effects of postoperative infectious complications extend beyond the short‐term outcomes.

Overall, the study of Matsuda et al. significantly contributed to the growing literature on the long‐term effects of postoperative infectious complications. However, conclusive evidence supporting the answer to their planned clinical question remains missing. That was likely due to the baseline differences between the patients with and without infectious complications, despite attempts to minimize the bias using statistical techniques. In this study, up to 10 clinicopathological factors differed between the two patient groups and all of them were not necessarily matched in the analysis. Due to these confounding factors, including unidentified factors, the results should be interpreted cautiously before confirming the causative relationship between postoperative infectious complications and long‐term survival outcomes. Despite some methodological adjustments, distinguishing between an association and causation is difficult without a supporting randomized clinical study. However, this is clinically impractical and unfeasible.

When reviewing basic research, multiple sets of evidence suggest that microbial infections cause various cytokine reactions and change the host's immune status significantly. This provides a pro‐tumor environment that facilitates tumor growth in organs, harboring minimal residual disease (MRD).^4^, ^5^ The proposed pathophysiology for tumor progression secondary to postoperative infections involves microbial pathogen‐associated molecular patterns directly affecting cancer growth. Another possible mechanism involves immunocompetent cells, which are activated in the setting of an infection. They are essential in accelerating the growth of cancer cells via humoral mediators, such as TNFa, IL‐1, IL‐6, IL‐18, the chemokine family, hepatocyte growth factor, and HMGB‐1. More importantly, immunocompetent cells suppress antitumor immunity via the upregulation of immunosuppressive cells, including regulatory T cells and myeloid‐derived suppressor cells.

To preserve a strict and professional attitude for assessment, the default paradigm should always be an “association” until proven otherwise. Thus, based on the current clinical evidence, the relationship between postoperative complications and impaired long‐term survival is more likely an association than causation. However, the occurrence of postoperative complications should be minimized since there may be a causative relationship based on basic research. Furthermore, future studies should determine the proportion of patients that would develop recurrence of malignant disease secondary to infectious complications. These initiatives may guide the development of new therapeutic options aimed at vulnerable populations. These may include utilizing agents to normalize cytokine signaling, immune checkpoint inhibitors to improve immune dysregulation in sepsis, and a liquid biopsy to detect MRD. Although many questions in this field remain unanswered, multidisciplinary approaches involving not only surgeons but other specialists such as oncologists, pathologists, molecular biologists, immunologists, and biostatisticians will open the door to the optimal treatment for patients with malignant tumors and infectious postoperative complications.

## CONFLICT OF INTEREST

Hideki Ueno is an Associate Editor of *Annals of Gastroenterological Surgery*. Hironori Tsujimoto is an Editorial Board Member of *Annals of Gastroenterological Surgery*. The authors declare no other conflicts of interests for this article.

## ETHICS STATEMENT

Approval of the research protocol: N/A.

Informed Consent: N/A.

Registry and the Registration No. of the study/trial: N/A.

Animal Studies: N/A.

## References

[1] Nowacki MP , Szymendera JJ . The strongest prognostic factors in colorectal carcinoma. Surgicopathologic stage of disease and postoperative fever. Dis Colon Rectum. 1983;26:263–8. 6839898 10.1007/BF02562495

[2] Varty PP , Linehan IP , Boulos PB . Intra‐abdominal sepsis and survival after surgery for colorectal cancer. Br J Surg. 1994;81:915–8. 8044621 10.1002/bjs.1800810641

[3] Matsuda A , Maruyama H , Akagi S , Inoue T , Uemura K , Kobayashi M , et al. Do postoperative infectious complications really affect long‐term survival in colorectal cancer surgery? A multicenter retrospective cohort study. Ann Gastroenterol Surg. 2022;7:110–20. 10.1002/ags3.12615 PMC9831895 36643360

[4] Tsujimoto H , Kobayashi M , Sugasawa H , Ono S , Kishi Y , Ueno H . Potential mechanisms of tumor progression associated with postoperative infectious complications. Cancer Metastasis Rev. 2021;40:285–96. 33389285 10.1007/s10555-020-09945-z

[5] Kouzu K , Tsujimoto H , Kishi Y , Ueno H , Shinomiya N . Role of microbial infection‐induced inflammation in the development of gastrointestinal cancers. Medicines (Basel). 2021;8:45. 34436224 10.3390/medicines8080045 PMC8400127

